# Primary renal mucinous adenocarcinoma masquerading as a giant renal cyst: a case report

**DOI:** 10.3389/fonc.2023.1129680

**Published:** 2023-05-08

**Authors:** Hong Zeng, Mengni Zhang, Yandong Xie, Minghao Wang, Jindong Dai, Xudong Zhu, Yuhao Zeng, Nanwei Xu, Peng Huang, Jinge Zhao, Guangxi Sun, Hao Zeng, Pengfei Shen

**Affiliations:** ^1^ Department of Urology, Institute of Urology, West China Hospital, Sichuan University, Chengdu, Sichuan, China; ^2^ Department of Pathology, West China Hospital, Sichuan University, Chengdu, Sichuan, China; ^3^ Department of Medical Oncology, Cancer Center, West China Hospital, Sichuan University, Chengdu, Sichuan, China

**Keywords:** mucinous adenocarcinoma, renal parenchyma, diagnosis, postoperative treatment, follow-up, case report

## Abstract

Mucinous adenocarcinoma of the kidney is rarely reported in the literature. We present a previously unreported mucinous adenocarcinoma arising from the renal parenchyma. A 55-year-old male patient with no complaints showed a large cystic hypodense lesion in the upper left kidney on contrast-enhanced computed tomography (CT) scan. A left renal cyst was initially considered, and a partial nephrectomy (PN) was performed. During the operation, a large amount of jelly-like mucus and bean-curd-like necrotic tissue was found in the focus. The pathological diagnosis was mucinous adenocarcinoma, and further systemic examination revealed no clinical evidence of primary disease elsewhere. Then the patient underwent left radical nephrectomy (RN), and the cystic lesion was found in the renal parenchyma, while neither the collecting system nor the ureters were involved. Postoperative sequential chemotherapy and radiotherapy were administered, and no signs of disease recurrence were observed over 30 months of follow-up. Based on a literature review, we summarize the lesion with rarity and the associated dilemma in preoperative diagnosis and treatment. Given the high degree of malignancy, a careful history analysis accompanied by dynamic observation of imaging and tumor markers is recommended for the diagnosis of the disease. Comprehensive treatment based on surgery may improve its clinical outcomes.

## Introduction

Mucinous adenocarcinoma is a class of malignant tumors characterized by the secretion of massive mucus and the microscopic presence of extracellular mucus lakes, which may spread and transfer to the periphery with the mucus (1). Most mucinous adenocarcinomas are diagnosed at an advanced stage, presenting with elevated tumor burden, frequent lymph node metastases, and usually impaired response to adjuvant therapy (2). Although the exact mechanism of carcinogenesis is not yet clear, gene mutations such as KRAS in the RAS family and overexpression of mucins have been found to be associated with the development of mucinous adenocarcinoma (3, 4). As a distinct subtype, the disease is common in the colorectum, stomach and ovary, accounting for 10%-20%, 2.2%-6.8% and 2.4%-4.9% of all primary tumors, respectively (3, 5, 6). Mucinous adenocarcinoma occurring in the kidney is fairly rare, as only a few dozen isolated cases have been reported previously, all of which were found to be located in the renal pelvis (7). Experience and knowledge of renal mucinous adenocarcinoma are extremely limited, and they have not been recognized in the 2022 World Health Organization (WHO) classification of renal tumors (8). This article described an unreported case of renal parenchymal mucinous adenocarcinoma from the whole process of diagnosis, treatment and follow-up in detail to provide ideas for the management of cystic lesions with uncertain nature.

## Case presentation

**Figure 1:**
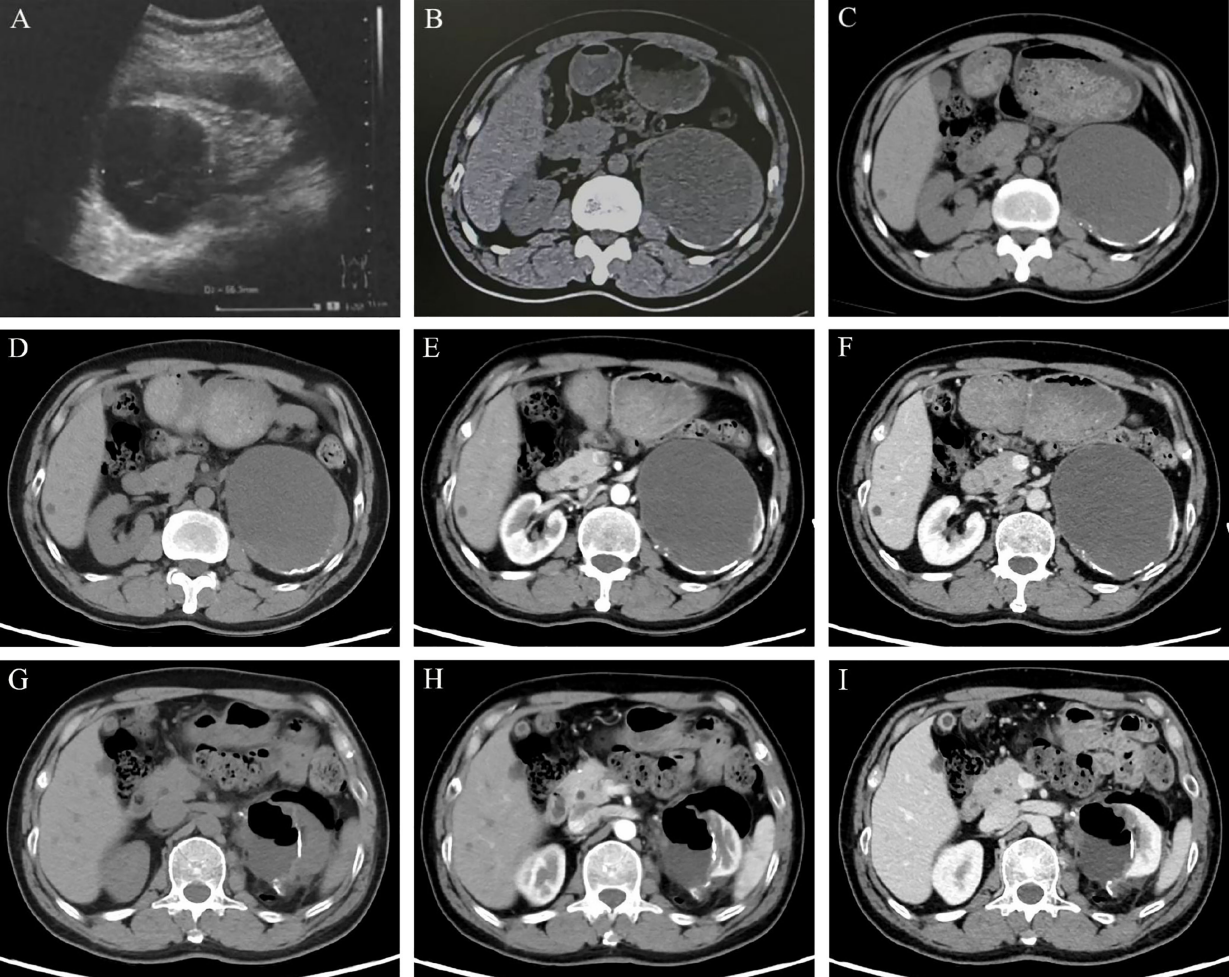
Imaging features of the left renal lesion. (**A**. February 28, 2013) Ultrasound revealed the cystic echogenic mass measuring 6.4 × 5.5 cm. (**B**. April 1, 2017) CT scan showed the cystic lesion of 9.5 × 7.6 × 6.7 cm. (**C**. October 22, 2019) CT scan showed the cystic lesion of 10.0 × 8.2 cm in cross-section with partial cyst wall calcification. Dynamic contrast-enhanced CT was performed to show the features of the lesion in UP **(D, G)**, CMP **(E, H)**, and NP **(F, I)**. (**D–F** on May 28, 2020) A mixed-density mass dominated by low density was found in the left kidney, with a cross-section of 10.3 × 8.5 cm and multiple high-density patches at the edge. (**G–I** on June 15, 2020) With localized encapsulated gas and effusion, the left kidney was partially absent accompanied by marginal densification. Abbreviations: UP, unenhanced phase; CMP, corticomedullary phase; NP, nephrographic phase.

A 55-year-old man presented with a large cystic mass of the left kidney without any subjective discomfort. Fifteen years before admission, he was diagnosed with a left renal cyst and subsequently underwent three paracenteses combined with anhydrous alcohol sclerotherapy. Thereafter, he received regular color Doppler ultrasound or computed tomography (CT) examinations. The objective data showed the cystic lesion measuring 6.4 × 5.5 cm in 2013, 9.5 × 7.6 cm in 2017, and 10.0 × 8.2 cm in 2019 with partial cyst wall calcification (**Figures 1A–C**). He had a medical history of blood transfusion due to gastric perforation 31 years earlier and a 13-year history of multiple hepatic cysts. Physical examination and routine blood tests revealed no abnormalities.

**Figure 2:**
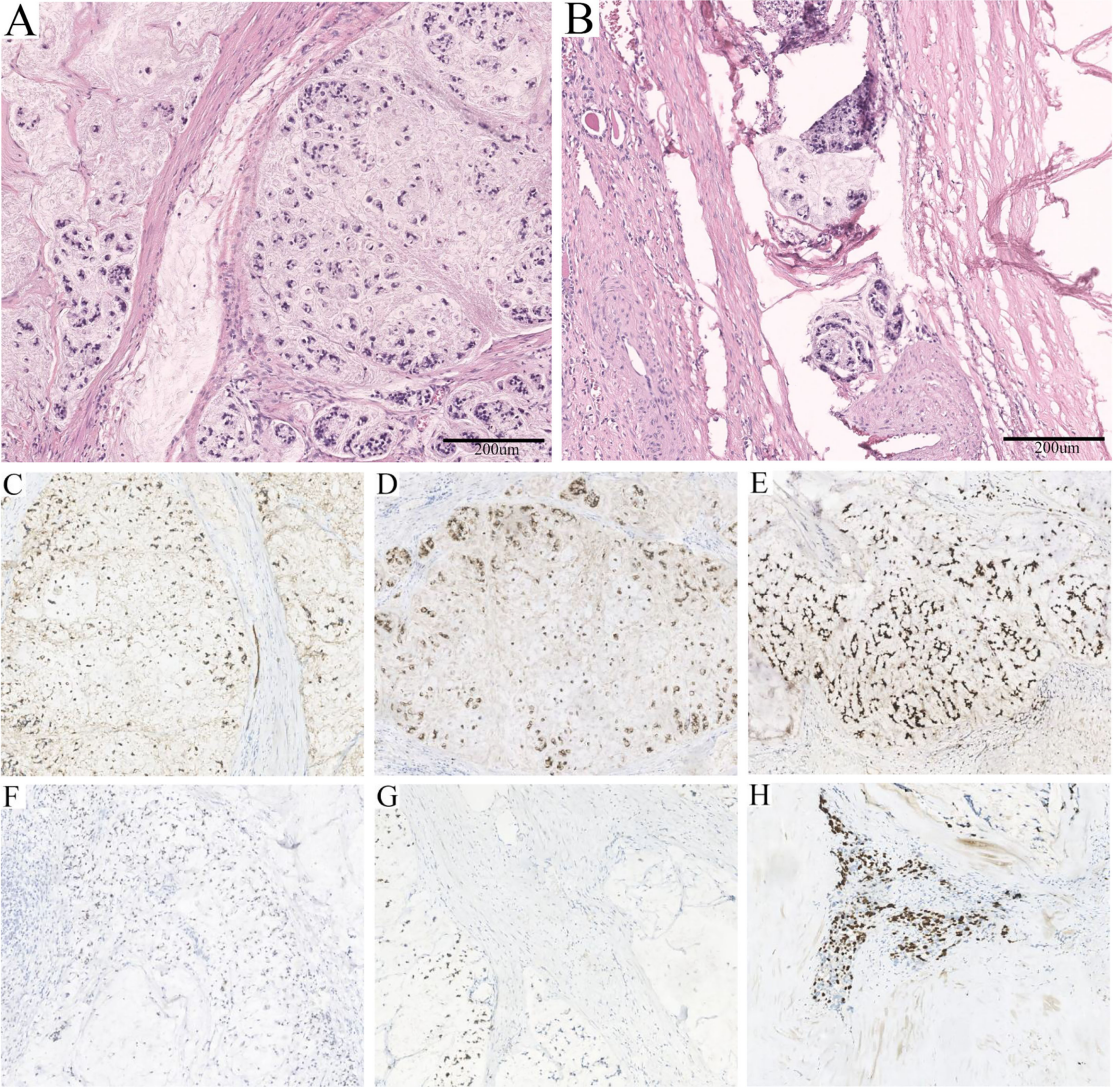
Pathological features of the lesion. **(A)** Massive mucus was found in the fibrocyst wall, and the tumor cells were scattered or glandular in arrangement (HE, ×100). **(B)** A few mucinous adenocarcinoma cells with multiple mucinous foci and scattered calcifications were found in the cystic wall (HE, ×100). **(C)** Strong positivity for PCK (×100). **(D)** Strong positivity for Villin (×100). **(E)** Strong positivity for CDX2 (×100). **(F)** Partial positivity for SATB2 (×100). **(G)** Weak positivity for Syn (×100). **(H)** Fifty percent positivity for Ki67 (×100).

An admission dynamic contrast-enhanced CT revealed a mixed-density mass dominated by low density in the left kidney, measuring 10.3 × 8.5 cm in cross-section, with multiple high-density patches at the rim (**Figures 1D-F**, **eFigure 1**). No distinct change in mass density was found during the phase of enhancement, and the imaging impression was a complex cyst (Bosniak classification grade IIF) (9). Laparoscopic partial nephrectomy (PN) of the left kidney was then performed. Intraoperative inspection revealed the cystic mass occupying the upper pole of the left kidney, with an uneven surface and adhesion to surrounding fat. When a minor incision of about 0.5 cm was made, a large quantity of jelly-like mucus and bean-curd-like necrotic tissue was found filling the lesion. On gross specimen examination, the removed cystoid tissue was approximately 9.2 × 6.2 × 1.8 cm in size and 0.2 × 0.9 cm in wall thickness, with a smooth outer wall and a rough inner wall. Microscopically, extensive mucus was found in the fibrocyst wall, and the tumor cells were scattered or glandular in arrangement (**Figure 2A**). A pathological diagnosis of mucinous adenocarcinoma was presented. Immunohistochemistry (IHC) demonstrated that the tumor cells were positive for PCK, Villin, CDX2, SATB2 (some cells), Syn (a few cells), and Ki67 (50%) but negative for CgA, Napsin A, TTF-1, GATA-3, CK7, PAX-8, CA9, RCC, and P63. (**Figures 2C–H**, **eFigure 2**).

The patient underwent further general examination. Painless gastroscopy and colonoscopy revealed no abnormal lesions, while the carbohydrate antigen 19-9 (CA19-9), carbohydrate antigen 72-4 (CA72-4), and carcinoembryonic antigen (CEA) indexes were elevated (79.20 U/ml, 50.10 U/ml, 5.24 ng/ml, respectively). A CT scan of the head and chest showed no evidence of metastasis, and a repeated abdominal CT scan revealed a localized encapsulation of gas and effusion (**Figures 1G–I**).

**Figure 3:**
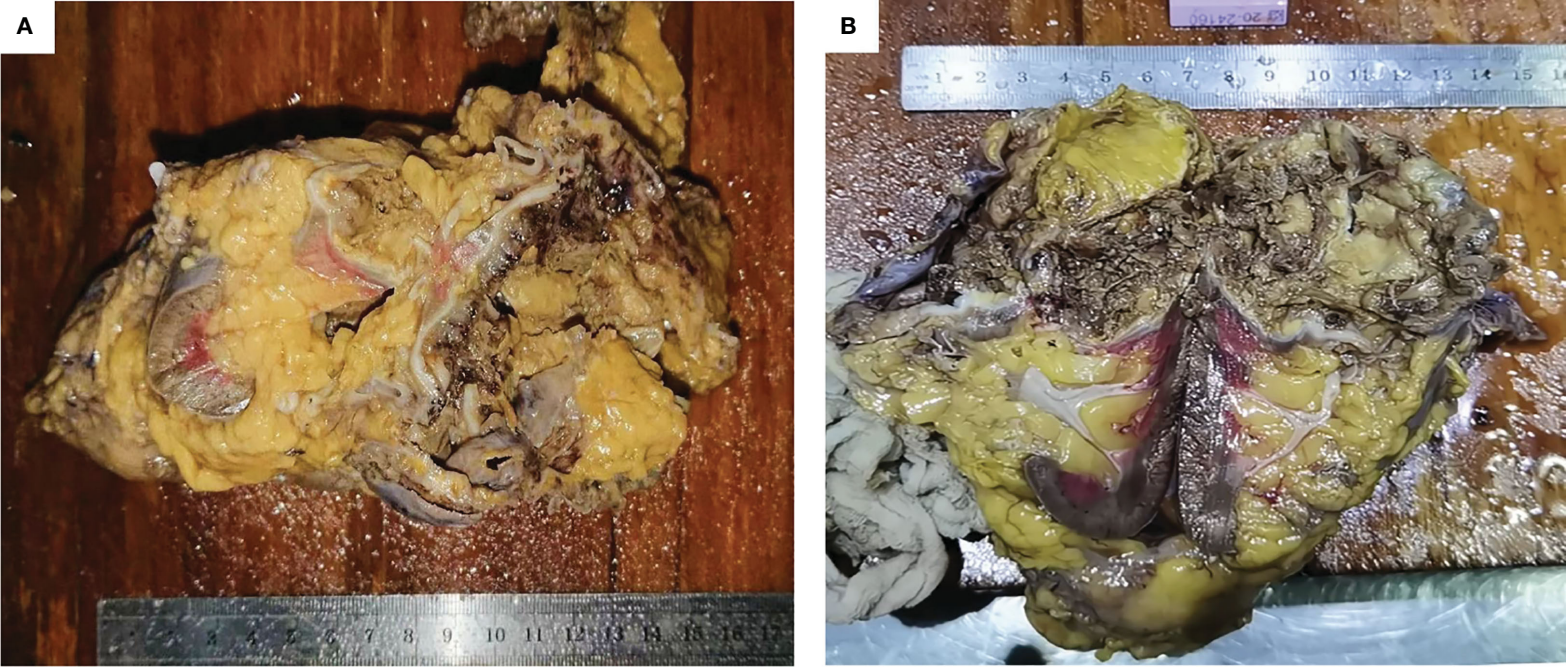
General manifestation of the specimen. **(A, B)** The cystic tumor was seen under the renal capsule and the cut surface was gray, with necrotic tissue.

Subsequently, laparoscopic radical nephrectomy (RN) of the left kidney was performed on June 19, 2020, during which dense adhesions with adjacent organs were observed. In gross specimen examination, a cystic mass of approximately 6.0 × 5.0 × 5.0 cm was seen immediately within the renal capsule with no visible invasion of the collecting system (**Figure 3**). The cut surface of the tumor was gray, with hemorrhage and necrotic tissue invading the capsule. Histopathological examination demonstrated fibrous cystic tissue in the renal parenchyma (**Figure 2B**). A few mucinous adenocarcinoma cells with multiple mucinous foci and scattered calcifications were found in the cystic wall. No tumor involvement was observed in the ureteral stump. Based on the above examinations, intraoperative and pathological findings, the final diagnosis of mucinous adenocarcinoma derived from the left renal parenchyma was proposed.

**Figure 4:**
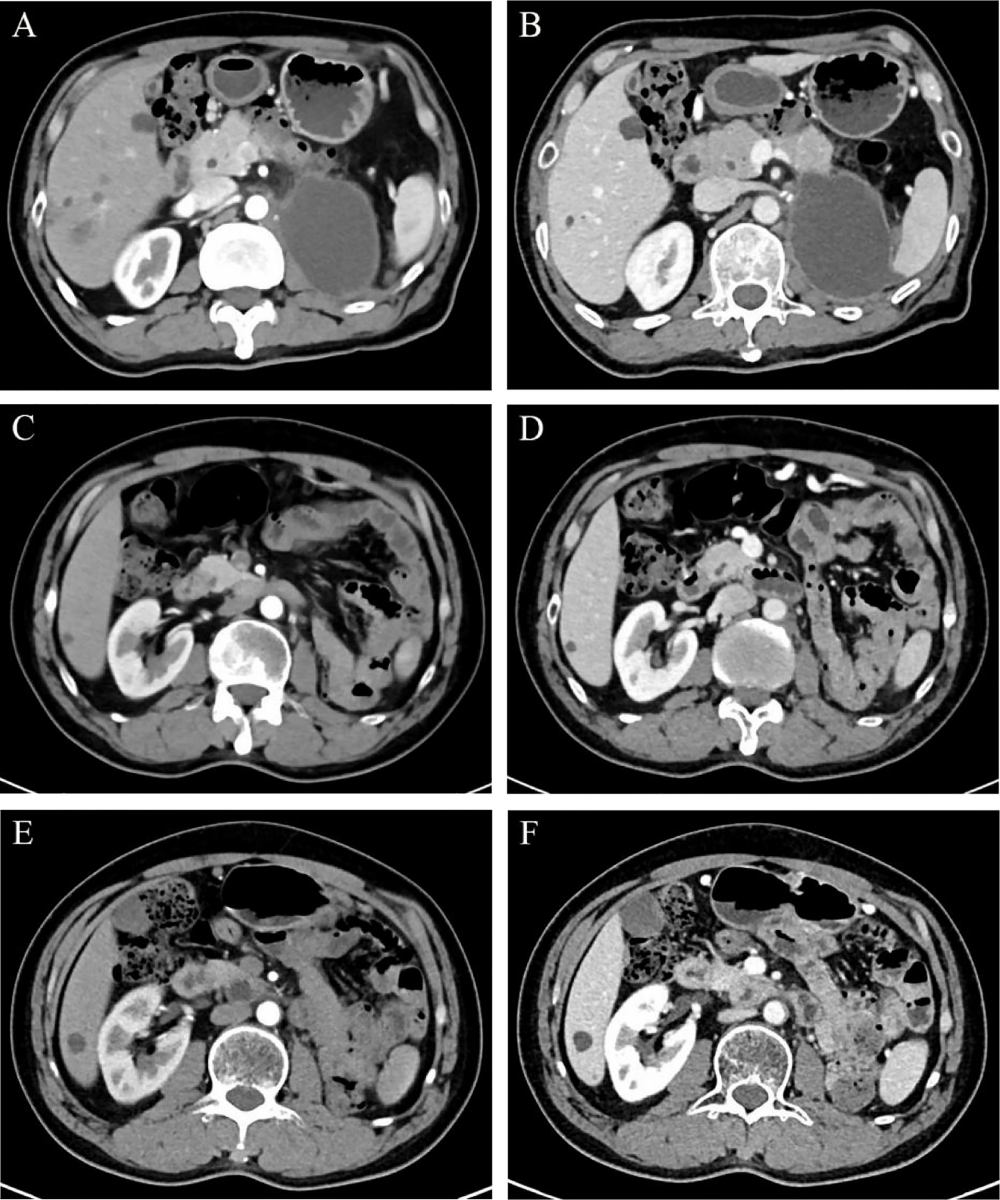
Regular dynamic contrast-enhanced CT scan manifestations in CMP **(A, C, E)** and NP **(B, D, F)**. (**A, B** on July 24, 2020) An 8.0 × 5.7 cm cystic low-density lesion was found in the original left renal area. (**C, D** on August 2, 2021) The cystic effusion was absorbed completely. (**E, F** on August 17, 2022) The original left kidney area was filled with intestines. Abbreviations: CMP, corticomedullary phase; NP, nephrographic phase.

One month after the radical surgery, a dynamic contrast-enhanced CT scan revealed a cystic lesion in the original left kidney area measuring 8.0 × 5.7 cm in cross-section (**Figures 4A, B**). Multidisciplinary consultation suggested that the patient should be given salvage therapy. From July 2020 to May 2021, he received eight cycles of chemotherapy with the SOX (oxaliplatin plus S-1) regimen in the first cycle and then the XELOX (oxaliplatin plus capecitabine) regimen in cycles 2-8 due to a general rash during oral S-1. After completion of chemotherapy, he was then subjected to 28 local radiotherapies at the original left renal area delivered by volumetric modulated arc therapy (VMAT) with a total dose of 5600 cGy in 200 cGy per fraction.

Periodic dynamic contrast-enhanced CT scans showed a gradual reduction in the cystic lesion in the left renal region, and a re-examination on August 2, 2021, showed that the lesions had disappeared (**Figures 4C, D**). Until this report, the patient remained stable with outpatient follow-up for 30 months and no evidence of recurrence (**Figures 4E, F**). The levels of the tumor markers gradually decreased after subsequent treatments during follow-up (**eFigure 3**). The timeline of interventions and outcomes for the patient is presented in **eFigure 4**.

## Discussion

Generally, mucinous adenocarcinoma occurs more frequently in the gastrointestinal tract, while the finding of mucinous adenocarcinoma in the urinary system is unexpected (10). Moreover, renal mucinous adenocarcinoma appears to be insidious and easily confused with simple renal cysts. For the discovery of mucinous adenocarcinoma in the kidney, normally, secondary tumor involvement from the surrounding organs would be our initial inference. Only cases of mucinous adenocarcinoma occurring in the renal pelvis have been previously reported, and the knowledge of the renal parenchyma origin remains few (7). The former invaded the collecting system and presented with abdominal pain, hydronephrosis, or calculi, while the latter showed cystic lesions on imaging without apparent discomfort (11–14). Given the challenges in the diagnosis and treatment of renal mucinous adenocarcinoma, rational countermeasures warrant further exploration.

On imaging, the left kidney lesion of the patient presented a gradual increase in volume without significant transformation in nature, which was inevitably always considered a benign cyst. Reviewing the literature, several cases of renal pelvis mucinous adenocarcinoma were indeed misdiagnosed as a renal cyst, resulting in a poor prognosis (13, 15). To avoid this, dynamic observation, comparison, and application of suitable classification systems like the Bosniak classification are recommended. However, due to the effect of interreader variability and variable reported carcinoma rates, escalation or degradation of the Bosniak classification may occur, leading to different treatment strategies (9). For uncertain cystic lesions, it is more appropriate to add other imaging tests, including magnetic resonance imaging (MRI) and contrast-enhanced ultrasound (CEUS), to improve the detection of soft tissue and tumor microvasculature, respectively (16, 17). Malignancy should not be ruled out in the presence of abnormal signs on imaging such as calcification, bleeding, and change in lesion density.

Although tumor markers are not routinely used as a screening tool for identifying renal malignancy, they may be a useful complement to detect the presence of mucinous adenocarcinoma. Abnormalities in CEA or CA19-9 have been reported in mucinous adenocarcinoma of the renal pelvis, but this accounts for only approximately 20% of patients (7, 18). A slight elevation of CA19-9 was found in a case of prostate mucinous adenocarcinoma (19). Moreover, elevated CEA was discovered in bladder-derived mucinous adenocarcinoma (20). CA72-4 was only increased in a female mucinous adenocarcinoma located at the bladder outlet and urethra, in which CEA, CA19-9, and CA12-5 were normal (21). In our case, elevated CEA, CA19-9, and CA72-4 were found simultaneously, reflecting the high tumor activity. The postoperative levels of tumor markers gradually decreased and remained stable, which was consistent with the imaging examination results. Therefore, monitoring the tumor markers could not only improve the accuracy of diagnosis but also serve as a reference index to evaluate disease progression. Nevertheless, the need for testing should be considered based on the Bosniak classification of the lesion and may be recommended for patients with grade IIF or higher.

Pathological examination is indispensable in the process of detecting mucinous adenocarcinoma, and the stage of diagnosis affects the prognosis. The presence of jelly-like mucus and necrotic tissue in the cystic lesion may be a characteristic manifestation, which has also been reported in cases of mucinous adenocarcinoma arising from the renal pelvis and ejaculatory duct (13, 14, 22). The definitive diagnosis was based on the demonstration of fibro-cystic tissue containing mucinous adenocarcinoma within the renal parenchyma without tumor invasion of the collecting system or ureter. Additionally, the identification of tumor sources depends on IHC. Negative Napsin A, TTF-1, and GATA-3 indicated that the tumor was not of pulmonary, thyroid, or urothelial origin (23, 24). The expression of Villin and CDX2 was confirmed, and the latter has also been reported in the majority of renal pelvic mucinous adenocarcinomas (7, 13, 14, 25). SATB2, a marker recognized to be highly sensitive and specific for colorectal carcinoma (CRC), was negative in cases of genitourinary mucinous adenocarcinoma, while our case was positive (7, 22, 26). Despite this, no suspicious lesions were ultimately found to support the involvement of CRC or other organ-derived tumors. Nevertheless, careful inspection is still necessary, as it facilitates the determination of the diagnosis and stage of the disease so that appropriate interventions can be developed.

To date, the etiology of renal mucinous adenocarcinoma is unknown. Congenital malformations, such as the horseshoe kidney, were thought to be involved in the tumor pathogenesis (13). Prolonged inflammation and infection of the cyst may be high-risk factors (12, 14, 27). A reasonable assumption is that the renal tubules may be induced to develop adeno-epithelial metaplasia and progress to adenocarcinoma, which requires more detailed research. Judging from the findings during surgery and the high expression rate of Ki67 in IHC, renal mucinous adenocarcinoma is characterized by strong invasiveness and a high degree of malignancy. In the absence of standard treatment, a more aggressive intervention is recommended. In the present case, it cannot be excluded that a few mucus entered the surgical field during the operation of left PN, which was also the cause of the subsequent remedial treatment. Therefore, as with the common renal cell carcinoma (RCC), RN is preferred in the patient with localized disease. Compared to open RN, minimally invasive procedures such as laparoscopic RN have been shown in large cohort studies to be associated with improved overall survival and reduced need for blood transfusions, postoperative pain, and length of hospitalization (28, 29).

Given the extreme rarity of renal mucinous adenocarcinoma, experience in treating mucinous adenocarcinoma of other sites can provide us with a reference. Previous studies have shown that the prognosis of mucinous adenocarcinoma is generally worse than that of nonmucinous adenocarcinoma, particularly those of colorectal and ovarian origin (3, 5, 30). The median overall survival (OS) for colorectal mucinous adenocarcinoma is only 8.0-14.0 months, and the commonly used chemotherapy regimens are XELOX and FOLFOX-4 (folinic acid, 5-fluorouracil (5-FU) and oxaliplatin, respectively) (31, 32). For advanced mucinous ovarian cancer, with a median survival of 15 months, platinum-based (e.g., oxaliplatin) combination chemotherapy is the usual regimen, but the response rate is relatively poor (33, 34). The 5-year survival rate for gastric mucinous adenocarcinoma is 42.8%-56.6%, and the efficacy of postoperative chemotherapy is unclear, primarily with generic 5-FU-based or platinum-based regimens (6, 35, 36). Even less experience was found in other rare sites of mucinous adenocarcinoma, where interleukin-2 (IL-2) and ubenimex were tried in mucinous adenocarcinoma of the renal pelvis with no disease recurrence at 14 months of postoperative follow-up (13). In addition, a patient with prostatic mucinous adenocarcinoma underwent radiotherapy after biochemical failure and did not relapse at a 4-year follow-up (37). As is well known, XELOX and SOX are chemotherapy regimens commonly used in gastrointestinal cancer, and this patient also received both schemes followed by local radiotherapy (3, 38). Until this report, no evident signs of disease recurrence were found, which may indicate the effectiveness of chemotherapy and radiation on the tumor.

## Conclusion

In brief, primary renal parenchymal mucinous adenocarcinoma is an extremely rare tumor without specific clinical, accurate diagnosis, and postoperative adjuvant therapies according to multidisciplinary consultation could warrant its prognosis.

## Data availability statement

The raw data supporting the conclusions of this article will be made available by the authors, without undue reservation.

## Ethics statement

Written informed consent was obtained from the individual(s) for the publication of any potentially identifiable images or data included in this article.

## Author contributions

HoZ, MZ, and YX composed the manuscript and literature review. MW, JD, and XZ collected the case details. YZ and NX were involved in the editing and collation of the images. PH contributed the part of radiotherapy. MZ contributed the part of pathology. JZ, GS, and HaZ revised the manuscript. PS censored and revised the article critically. All authors contributed to the article and approved the submitted version.
