# Supplementary material for: Primary renal mucinous adenocarcinoma masquerading as a giant renal cyst: a case report
Source: Front Oncol. 2023 May 8;13:1129680. doi: 10.3389/fonc.2023.1129680 (PMC10200912; doi:10.3389/fonc.2023.1129680)
Supplement: Supplementary file 1 [file DataSheet_1.pdf]

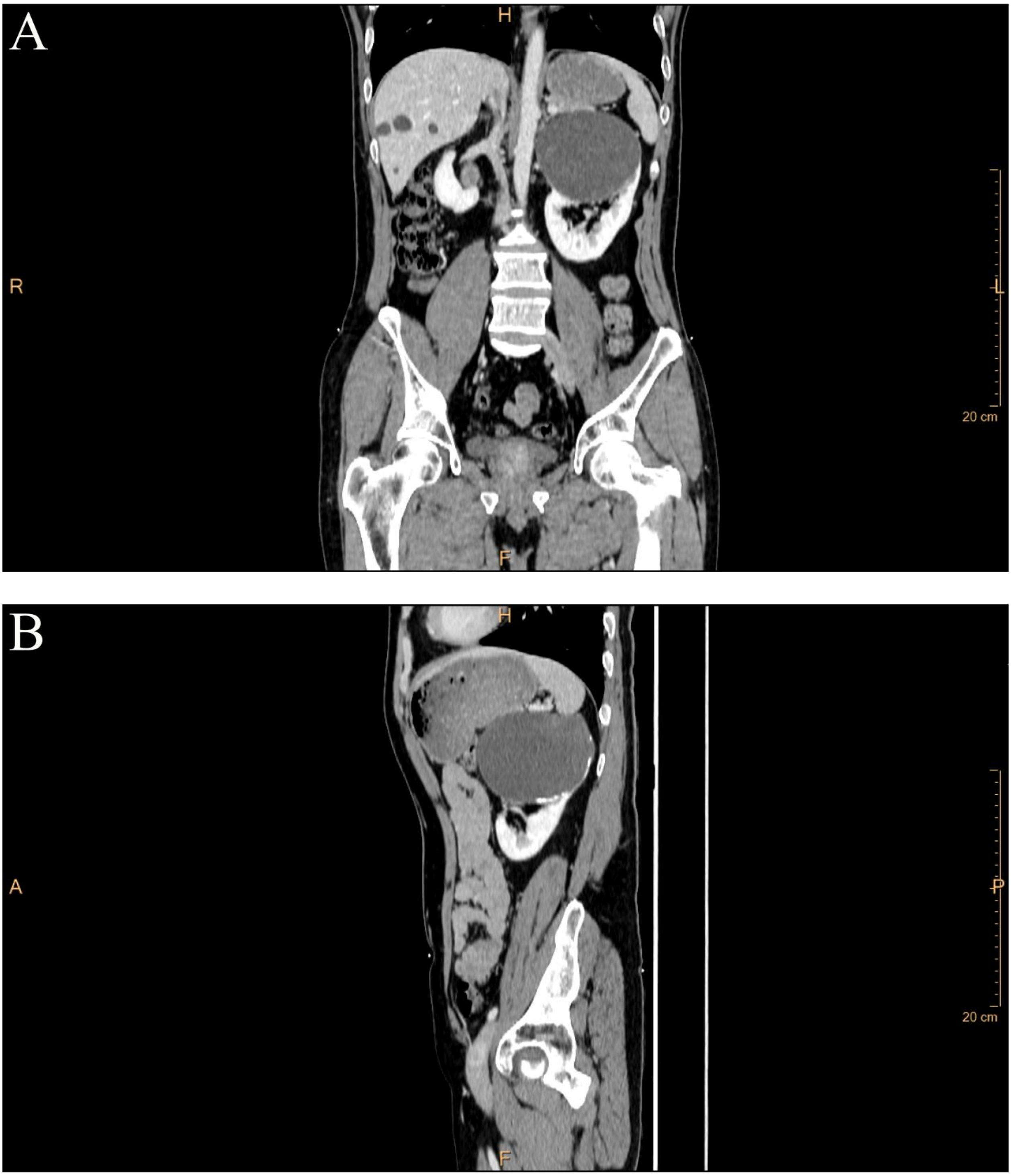

eFigure 1 Imaging features of the left renal lesion in coronal and sagittal planes. (**A May 28, 2020**) Coronal imaging of the lesion in NP. (**B May 28, 2020**) Sagittal imaging of the lesion in NP. Abbreviations: NP, nephrographic phase; H, head; F, foot; L, left; R, right; A, anterior; P, posterior.
