# Supplementary material for: Primary renal mucinous adenocarcinoma masquerading as a giant renal cyst: a case report
Source: Front Oncol. 2023 May 8;13:1129680. doi: 10.3389/fonc.2023.1129680 (PMC10200912; doi:10.3389/fonc.2023.1129680)
Supplement: Supplementary file 2 [file DataSheet_2.pdf]

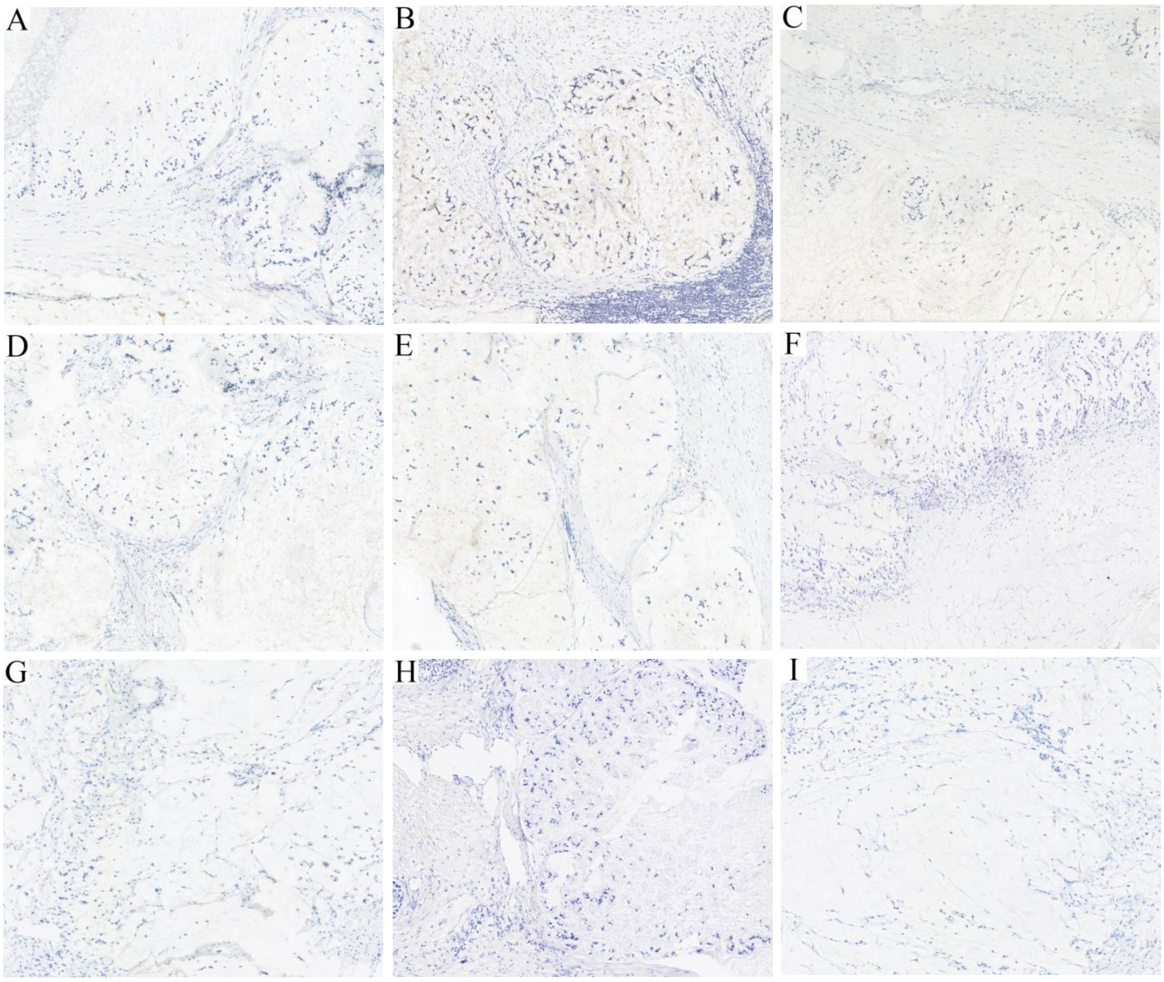


**eFigure 2** Immunohistochemistry reults of the lesion. **(A)** Negative for CgA (×100). **(B)** Negative for Napsin A (×100). **(C)** Negative for TTF-1 (×100). **(D)** Negative for GATA-3 (×100). **(E)** Negative for CK7 (×100). **(F)** Negative for PAX-8 (×100). **(G)** Negative for CA9 (×100). **(H)** Negative for RCC (×100). **(I)** Negative for P63 (×100).
