# Supplementary figures and images for: Primary renal mucinous adenocarcinoma masquerading as a giant renal cyst: a case report
Source: Front Oncol. 2023 May 8;13:1129680. doi: 10.3389/fonc.2023.1129680 (PMC10200912; doi:10.3389/fonc.2023.1129680)

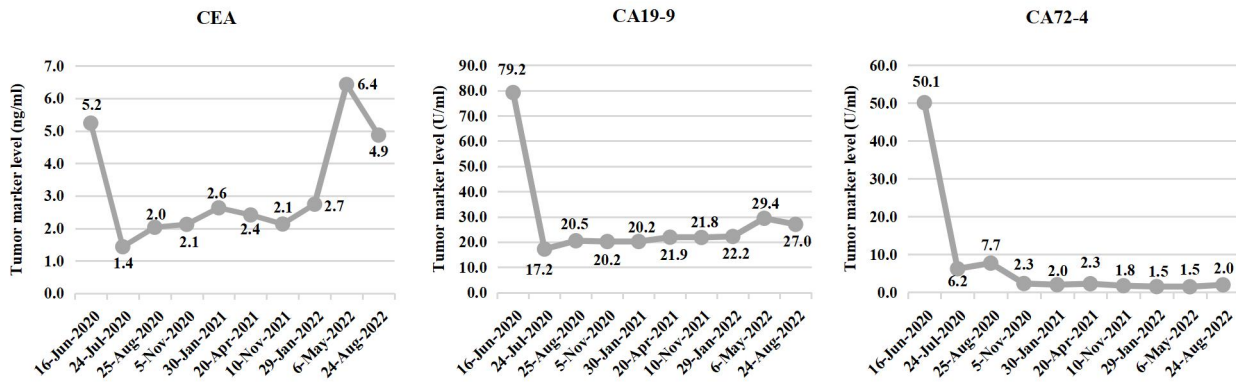

**eFigure 3** Trends in CEA, CA19-9, and CA72-4 of the patient.

Supplement: Supplementary file 3 [file DataSheet_3.pdf]
