# Supplementary material for: Primary renal mucinous adenocarcinoma masquerading as a giant renal cyst: a case report
Source: Front Oncol. 2023 May 8;13:1129680. doi: 10.3389/fonc.2023.1129680 (PMC10200912; doi:10.3389/fonc.2023.1129680)
Supplement: Supplementary file 4 [file DataSheet_4.pdf]

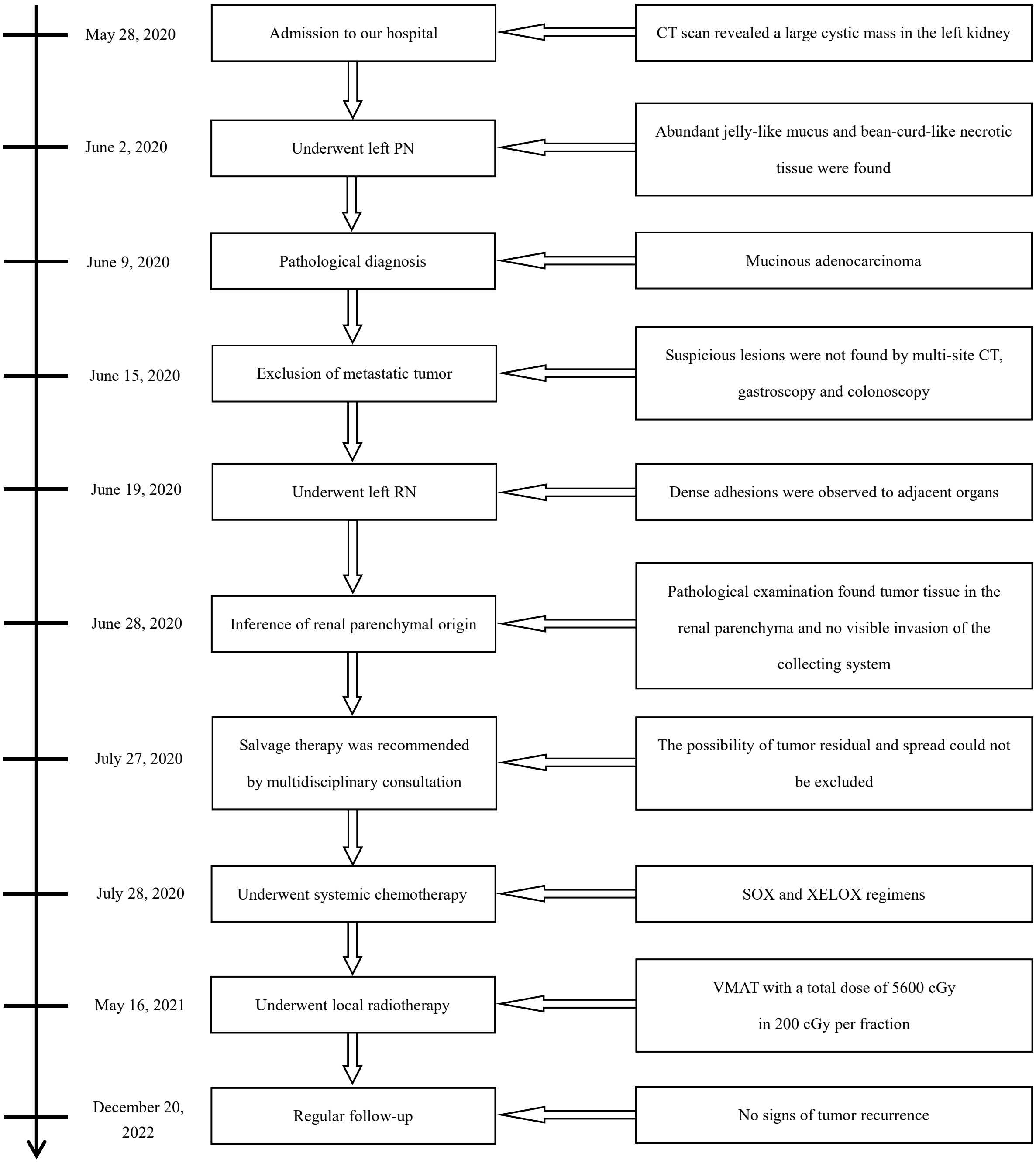

eFigure 4 Timeline of interventions and outcomes. Abbreviations: CT, computed tomography; PN, partial nephrectomy; RN, radical nephrectomy; VMAT, volumetric modulated arc therapy.
